# A Single Nucleotide Polymorphism in Catalase Is Strongly Associated with Ovarian Cancer Survival

**DOI:** 10.1371/journal.pone.0135739

**Published:** 2015-08-24

**Authors:** Jimmy Belotte, Nicole M. Fletcher, Mohammed G. Saed, Mohammed S. Abusamaan, Gregory Dyson, Michael P. Diamond, Ghassan M. Saed

**Affiliations:** 1 Department of Obstetrics and Gynecology, The C.S. Mott Center for Human Growth and Development, Wayne State University School of Medicine, Detroit, MI, United States of America; 2 Karmanos Cancer Institute, Detroit, MI, United States of America; 3 Department of Obstetrics and Gynecology, Georgia Regents University, Augusta, GA, United States of America; CNR, ITALY

## Abstract

Ovarian cancer is the deadliest of all gynecologic cancers. Recent evidence demonstrates an association between enzymatic activity altering single nucleotide polymorphisms (SNP) with human cancer susceptibility. We sought to evaluate the association of SNPs in key oxidant and antioxidant enzymes with increased risk and survival in epithelial ovarian cancer. Individuals (n = 143) recruited were divided into controls, (n = 94): healthy volunteers, (n = 18), high-risk *BRCA1/2* negative (n = 53), high-risk *BRCA1/2* positive (n = 23) and ovarian cancer cases (n = 49). DNA was subjected to TaqMan SNP genotype analysis for selected oxidant and antioxidant enzymes. Of the seven selected SNP studied, no association with ovarian cancer risk (Pearson Chi-square) was found. However, a catalase SNP was identified as a predictor of ovarian cancer survival by the Cox regression model. The presence of this SNP was associated with a higher likelihood of death (hazard ratio (HR) of 3.68 (95% confidence interval (CI): 1.149–11.836)) for ovarian cancer patients. Kaplan-Meier survival analysis demonstrated a significant median overall survival difference (108 versus 60 months, p<0.05) for those without the catalase SNP as compared to those with the SNP. Additionally, age at diagnosis greater than the median was found to be a significant predictor of death (HR of 2.78 (95% CI: 1.022–7.578)). This study indicates a strong association with the catalase SNP and survival of ovarian cancer patients, and thus may serve as a prognosticator.

## Introduction

Epithelial ovarian cancer (EOC) accounts for 85 to 90% of all cancers of the ovaries, fallopian tubes and primary peritoneum; and displays various histologies such as serous, mucinous, or endometrioid [1]. Ovarian cancer is the deadliest of all gynecologic cancers with an estimated 22,980 new cases and 14,270 deaths expected in 2014 in the US alone [1,2]. Typically, treatment of ovarian cancer is performed with either cytoreductive surgery (CRS) followed by platinum/taxane combination chemotherapy or neoadjuvant chemotherapy with interval CRS [3,4]. Generally, a 50–80% complete clinical response can be achieved in patients with advanced disease. Unfortunately, most treated patients will relapse within 18 months with chemoresistant disease [5]. While the chances of long-term patient survival are significantly increased when the cancer is detected at its early stage, to date, there is no reliable method available for early detection of this disease [5].

Epidemiologic studies have clearly established the role of family history as an important risk factor for both breast and ovarian cancers [6]. Mutations in *BRCA* are currently utilized to evaluate risk for breast and ovarian cancer, however, this method is not ideal because the mutations are so rare (1 out of 500 individuals), leading to a small overall impact on mortality rate [7]. Genomic variations between individuals have been increasingly used in the practice of medicine [8–12]. A single nucleotide polymorphism (SNP) occurs because of point mutations that are selectively maintained in populations and are distributed throughout the human genome at an estimated overall frequency of at least one in every 1000 base pairs [13]. Non-synonymous SNPs substitute encoded amino acids in proteins, and are more likely to alter the structure, function, and interaction of the protein [14]. Recent evidence demonstrates an association between enzymatic activity altering SNPs in oxidative DNA repair genes and antioxidant genes with human cancer susceptibility [15]. Additionally, a pro-oxidant state has been implicated in the pathogenesis of several malignancies, including ovarian cancer [16,17]. The current study is based on the fact that certain SNPs present in key oxidants and antioxidants enzymes result in altered enzymatic activity, as well as our previously published work delineating the role of oxidative stress in ovarian cancer. The goal of this study was to determine whether specific SNP in key oxidant and antioxidant enzymes are associated with the increased risk as well as overall survival of ovarian cancer patients.

## Materials and Methods

### Study design

We performed a case-control study comparing female subjects with and without EOC and determined whether there is an association with several selected SNPs in established redox genes. Eligible women were 19 to 80 years of age and were previously recruited through the Karmanos Cancer Institute’s Genetic Registry (KCIGR), Detroit, MI. Research activities and method of consent were conducted with the approval of Wayne State University Institutional Review Board (IRB#024199MP2F(5R)). Informed written consent forms were utilized and permission was granted for the collection of blood samples and for access to medical records for all subjects.

#### Patient Population

Recruited individuals (n = 143) were divided into controls (94), healthy volunteers (n = 18), high-risk *BRCA1/2* negative (n = 53), high-risk *BRCA1/2* positive (n = 23) and ovarian cancer cases (n = 49). Controls were selected primarily from research subjects, considered high-risk for breast and ovarian cancers, without ovarian cancer that underwent genetic screening for *BRCA1/2* carrier status. Of note, the criteria used for screening included personal history of breast and ovarian cancers, family histories of breast and ovarian cancers, and *BRCA1/2* mutations. Additionally, healthy volunteers were also recruited as controls from the metropolitan Detroit area with no such histories. Cases were selected based on histopathology-confirmed primary diagnosis of EOC. All participants, except healthy volunteers, had previously undergone *BRCA1/2* testing and the results were made available to us.

Samples used for this study were collected from participants recruited between 1999 and 2012. DNA samples were utilized to determine the presence of polymorphisms in the genes described in Table 1. The SNPs were chosen based on previously reported associations with several cancers [18–26]. Of the 143 subjects, 49 (34.3%) had a primary diagnosis of ovarian cancer while 94 (65.7%) without cancer served as controls. For the ovarian cancer cohort: 13 (26.5%) were *BRCA1/2* positive as compared to 34 (69.4%) *BRCA1/2* negative; 2 (4.1%) cases were missing. The data is normally distributed with the age of enrollment ranged from 18 to 90 with a mean of 52 ± 15 and a median age of 52. The age at diagnosis ranged from 23 to 77, with a mean of 52 ± 11 and a median age of 52. The racial distribution was 88.8% (Caucasian), 8.4% (African-American) and 2.8% (Other). Personal and family histories of breast cancer, ovarian cancer, other cancers, and *BRCA1/2* were quantified. The frequencies of the presence of the SNP (heterozygous plus homozygous mutant) compared to homozygous wild type were determined for each gene studied.

**Table 1:** Characteristics of single nucleotide polymorphisms examined for genotyping.

Gene (RS)	SNP	NCBI MAF	Chromosomal location	Known AA Switch	Effect on activity
*CAT* (rs1001179)	C-262T	0.123	11p13	Unknown	Decrease
*CYBA* (rs4673)	C242T	0.303	16q24.3	Tyr to His	Increase
*GPX1* (rs3448)	C-1040T	0.176	3p21.31	Unknown	Unknown
*GSR* (rs1002149)	G201T	0.191	8p12	Unknown	Unknown
*MnSOD* (rs4880)	T47C	0.371	6q25.3	Ala to Val	Decrease
*MPO* (rs2243828)	T-764C	0.23	17q22	Unknown	Decrease
*NOS2* (rs2297518)	C2087T	0.173	17q11.2	Ser to Leu	Increase

AA; amino acid, Ala; alanine, CAT; catalase, CYBA; NAD(P)H oxidase subunit (NOX4), GSR; glutathione reductase, GPX; glutathione peroxidase, His; histidine, Leu; leucine, MAF; minor allele frequency, MnSOD; manganese superoxide dismutase, MPO; myeloperoxidase, NCBI; National Center for Biotechnology Information, NOS2; nitric oxide synthase, Ser; serine, SNP; single nucleotide polymorphism, Tyr; tyrosine, Val; valine.

### Purification of DNA and the TaqMan SNP Genotyping Assay for SNPs

DNA, from blood samples, was isolated by the Applied Genomics Technology Center (AGTC, Detroit, MI). DNA was extracted with QIAamp DNA mini kit per the manufacturer’s protocol (Qiagen, Valencia, CA) [27]. The TaqMan SNP Genotyping Assay Sets (Applied Biosystems, Carlsbad, CA) (NCBI dbSNP genome build 37, MAF source 1000 genomes) were used to genotype selected SNPs described in Table 1. The AGTC performed this assay and analysis was done utilizing the QuantStudio 12K Flex Real-Time PCR System (Applied Biosystems).

### Statistical analysis

Data were analyzed using SPSS (IBM, Armonk, New York) for Mac V.22. The variables selected for the analyses include genotypes, age at diagnosis, and age at enrolment, personal and family histories of breast, ovarian, and *BRCA1/2* mutations, in addition to other malignancies. Using the median age at diagnosis/enrolment as a cut point, we dichotomized the “age at diagnosis” variable. The “race” variable was categorized as: Caucasian, African-American or Other. We consolidated the following tumor and clinical variables into binary categorical schemes: International Federation of Gynecology and Obstetrics (FIGO) stages into early (IIA-IIIB) and advanced (IIIC-IV); FIGO grades (G1/2) and (G3); histology (serous and other). For all the genes studied, the “genotype” variable was dichotomized using the following scheme: homozygous wild type versus homozygous mutant plus heterozygous mutant. To compare cases to controls on the selected demographic, clinical, and genotypic characteristics, we performed Pearson Chi-square analysis. The recurrence rate was determined as the percentage of patients that have gone into remission, but the disease has returned months or years later, based on physical examination, radiological studies and serum CA-125 levels.

#### Cox regression and Kaplan-Meier analyses of variables as a predictor of overall survival

To study the impact of the SNPs on overall survival, Cox regression analyses were performed using the above-listed variables and classification schemes, using the likelihood ratio forward stepwise method. Several method simulations were performed such as: forced entry (ENTER), forward LR (likelihood ratio), etc. The forward LR was chosen for the final analysis. This method is a stringent model that selects the strongest predictors of the outcome to be included in the final model. Table 2 includes the strongest predictors kept by the model as well as variables rejected by the model. All patients received the standard of care after tumor board discussion. Details on treatment characteristics were not available. Additionally, Kaplan-Meier survival curves were generated for the variables selected by the model. We conducted all analyses at p-value < 0.05 for statistical significance.

**Table 2:** Cox regression analysis for selected SNPs in key oxidants and antioxidants genes in ovarian cancer.

**Variables in the Equation**				
			95% CI for HR	
	Significance	HR	Lower	Upper
Age at Diagnosis > Mean	.045*	2.782*	1.022	7.578
*CAT* (CT+TT)	.028*	3.688*	1.149	11.836
**Variables Analyzed by Cox Regression but Rejected by the Model**.				
	Score	Significance		
Race (Caucasian)	.580	.446		
Stage (III-IV)	.708	.400		
Grade (High)	.708	.400		
*GSR* (CT+TT)	.411	.522		
*GPX* (CT+TT)	.000	.988		
*MnSOD* (CT+TT)	1.020	.312		
*NOS2* (CT+TT)	2.084	.149		
*CYBA* (CT+TT)	1.229	.268		
*MPO* (CT+TT)	.178	.673		
Histology (Serous)	1.016	.314		

Adjusted Hazard Ratio (HR) for the variables included in the model.

* p<0.05, degrees of freedom = 1 for all analyses. CYBA; NAD(P)H oxidase subunit (NOX4), GPX; glutathione peroxidase, GSR; glutathione reductase, MnSOD; manganese superoxide dismutase, MPO; myeloperoxidase, NOS2; inducible nitric oxide synthase. For this analysis, several “Method”simulations were performed such as: forced entry (ENTER), forward LR (likelihood ratio), etc. The forward LR was chosen for the final analysis. Table 4 includes the strongest predictors kept by the model as well as those rejected by the model. The Cox regression model generated the scores in the table. The P-values are noted in the column significance; *p<0.05, is considered statistically significant.

## Results

We performed side-by-side comparison between ovarian cancer cases and controls using Pearson chi-square analysis. Racial distribution was statistically similar between the groups (p>0.05). As expected, “personal or family history of ovarian cancer”, “personal or family history of other cancers” and “advanced age” were significantly different between the groups and are known ovarian cancer risk factors (p<0.05, Table 3). Comparative analyses for manganese superoxide dismutase (*MnSOD*, rs4880), NAD(P)H oxidase (*CYBA*, rs4673), glutathione peroxidase (*GPX1*, rs3448), inducible nitric oxide synthase (*NOS2*, rs2297518), myeloperoxidase (*MPO*, rs2243828), glutathione reductase (*GSR*, rs1002149), and catalase (*CAT*, rs1001179) did not find a significant difference between the cases and controls (Table 3). Out of the 49 ovarian cancer cases, 38 (77.5%) were further analyzed by the Cox regression method, and 11 (22.5%) were dropped due to missing data. The majority of the cases were serous histology, advanced stage, and high-grade tumors (Table 4). The recurrence rate was found to be 60.5%.

**Table 3:** Comparison of cases and controls based on demographic, personal or family history of cancer, and genotypic characteristics.

	Controls (%)	Ovarian Cancer (%)	P-value (Pearson Chi-square, 2-tailed)
**Age at enrolment (n = 125)**	n (76)	n (49)	<0.001*
< Median	51 (67.1)	8 (16.3)	
> Median	25 (32.9)	41 (83.7)	
**Race (n = 143)**	n (94)	n (49)	
Caucasian	81 (86.2)	46 (93.9)	
African-American	10 (10.6)	2 (4.1)	
Other	3 (3.2)	1 (2.0)	
**Personal / Family History of Cancer (Yes)**	n (94)	n (49)	
Breast (n = 105)	67 (71.3)	38 (77.6)	.480
Ovarian (n = 81)	33 (35.1)	48 (98.0)	<0.001*
*BRCA1/2* (n = 23)	18 (19.1)	5 (10.2)	.168
Other Cancer (n = 69)	39 (44.3)	30 (62.5)	.043*
**SNP (Yes)**			
*NOS2* (n = 49)	34 (37.4)	15 (30.6)	.424
*CYBA (n = 92)*	57 (60.6)	35 (71.4)	.201
*MPO* (n = 56)	36 (39.1)	20 (41.7)	.771
*GSR* (n = 36)	27 (30.3)	9 (19.6)	.180
*GPX* (n = 61)	40 (43.5)	21 (42.9)	.943
*CAT* (n = 49)	30 (31.9)	19 (38.8)	.412
*MnSOD* (n = 103)	66 (72.5)	37 (75.5)	.703

*p< 0.*05*, *CAT; catalase*, *CYBA; NAD(P)H oxidase subunit (NOX4)*, *GPX; glutathione peroxidase*, *GSR; glutathione reductase*, *MnSOD; manganese superoxide dismutase*, *MPO; myeloperoxidase*, *NOS2; inducible nitric oxide synthase*.

**Table 4:** Stage, grade and pathologic characteristics of the cancer cases.

Tumor Characteristics	Number (%)
**Stage (n = 38)**	
IA-IIIB	10 (26.3)
IIIC-IV	28 (73.7)
Total	38 (100)
**Grade (n = 38)**	
G1/2	6 (15.8)
G3	32 (84.2)
Total	38 (100)
**Histology (n = 38)**	
Serous	34 (89.5)
Clear Cell	1 (2.6)
Endometrioid	1 (2.6)
Total	38 (100)

### The *CAT* SNP is a predictor of shorter survival

**Fig 1:**
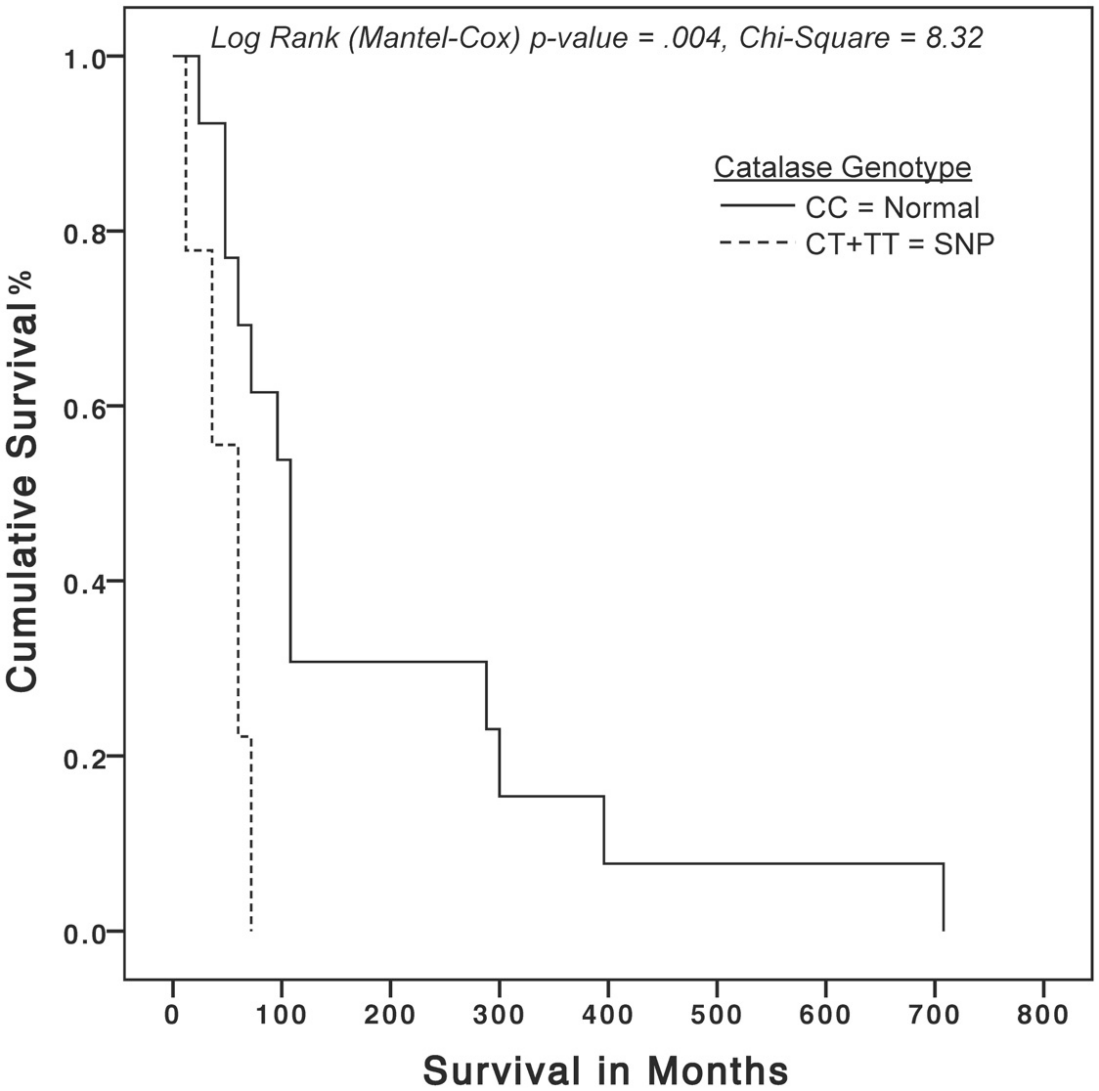
Kaplan-Meier overall survival curves for in ovarian cancer utilizing a specific catalase SNP. The solid curve represents cases with (CC) homozygous wild-type genotype as compared to the dashed curve, which represents cases with homozygous mutant plus heterozygous mutant (CT+TT) genotypes. The X-axis represents patient survival in months; the Y-axis represents cumulative survival percentage. Chi-square p-value 0<0.05 is considered statistically significant.

At the time of these analyses, there were 26 deaths (18.2%) and 117 (81.8%) subjects alive. Among the SNPs examined, only *CAT* (rs1001179) was identified as a predictor of shorter survival by the Cox regression model with a hazard ratio (HR) of 3.68 (95% CI: 1.149–11.836, p = 0.028) (Table 4A). As expected, “age at diagnosis” greater than the median (52) was found to be a significant predictor of death with an HR of 2.78 (95% CI: 1.022–7.578, p = 0.045) (Table 4). The variables selected for the analyses, but rejected by the model are listed in Table 4. Kaplan-Meier (K-M) survival analysis factored by *CAT* genotype, which used 84.6% of the deaths, demonstrated a statistically significant median overall survival difference (108 [95% CI: 79–137] versus 60 [95% CI: 40–80] months, p<0.05) and a mean overall survival difference (182 [95% CI: 75.5–288] versus 47 months [95% CI: 31–60], p<0.05) for subjects with the normal genotype as compared to the *CAT* SNP genotype (Fig 1).

## Discussion

A large body of evidence suggests that ovarian cancer patients have decreased levels of circulating antioxidants and higher levels of oxidative stress [16,17,28–32]. We have reported the existence of a persistent pro-oxidant state in EOC that included increased expression of key pro-oxidant enzymes such as inducible nitric oxide synthase (iNOS), NAD(P)H oxidase, and MPO [16,32,33]. Interestingly, the expression of MPO in EOC cells and tissues came as a surprise as it is an oxidant-generating enzyme typically found in cells of myeloid origin [34]. We have also determined that MPO can produce the nitrosonium cation (NO^+^) utilizing NO produced by iNOS. This is important because NO^+^ causes s-nitrosylation of caspase-3, and inhibition of its activity, resulting in a decrease in apoptosis [32]. This mechanism further explains the observation that EOC cells manifest significantly decreased apoptosis and increased survival [32,33,35,36]. Interestingly, the evaluation of mutations in the various redox enzymes in the form of SNPs is an active area of scientific research [37–45]. Genetic polymorphisms are known to be associated with cancer susceptibility and can be determined by studying functional polymorphisms in genes that control the levels of cellular reactive oxygen species and oxidative damage, including SNPs for genes involved in carcinogen metabolism (detoxification and/or activation), antioxidants, and DNA repair pathways [46]. For example, germline mutations in *BRCA1* or *BRCA2* are associated with ovarian cancer at a rate of only 20–40%, suggesting the presence of other unidentified mutations in other genes as an etiology [14,47,48]. Additional genetic variations, many of which have been identified in recent genome-wide association studies (GWAS), have been hypothesized to act as low to moderate penetrant alleles, which contribute to ovarian cancer risk, as well as other diseases [14,49]. In support of this, recent studies have also associated genetic polymorphisms in genes involved in suppression of tumorigenicity as well as those involved in cell cycle with ovarian cancer [50,51].

For this study, we sought to evaluate the association of specific SNPs in key oxidant and antioxidant enzymes with increased risk and overall survival of ovarian cancer. The analysis of the patient population revealed that the average age at diagnosis and racial distribution of those diagnosed with ovarian cancer were consistent with known risk factors for ovarian cancer, specifically, women of North American decent and those over 50 years old. Currently we demonstrated that there is no association between the selected SNPs and risk of developing ovarian cancer (Table 2). It is important to emphasize the fact that although the selected SNPs for this study were not found to be associated with ovarian cancer risk, additional change of function SNPs for these enzymes exist and should be explored further. Of the SNPs studied when examining survival, we found the *CAT* SNP (rs1001179) to be a significant predictor of death when present in ovarian cancer patients as illustrated by the Cox regression and K-M survival analyses (Table 3 and Fig 1). Specifically, ovarian cancer patients with the *CAT* SNP died significantly sooner than those without it (Fig 1). The *CAT* SNP (rs1001179) is found in the promoter region of the *CAT* gene, substituting allele C with T at position -262 in the 5’ region of chromosome 11 and is correlated with decreased enzyme activity level [52]. Catalase is a very important and ubiquitous enzyme involved in the degradation of two molecules of hydrogen peroxide (H_2_O_2_) to water and oxygen. The current findings are consistent with several other studies, which linked this specific SNP with risk, response to adjuvant treatment and survival of cancer patients [18,19,24,53]. Specifically, low serum CAT levels were associated with adverse prognosis for ovarian cancer [21]. Our data provides a possible explanation for low serum CAT levels, which may be a result of a *CAT* SNP that lowers enzymatic activity. Moreover, mechanistic studies have identified H_2_O_2_, a result of oxidative stress, enhance angiogenesis and tumor invasiveness through several pathways including: hypoxia inducible factor 1-alpha, p38 MAPK and snail [54,55]. It appears that the final common pathway culminates to epidermal growth factor (EGF)-induced down-regulation of epithelial cadherin expression that can be inhibited by exogenous CAT [54]. Epithelial-cadherin is a cell-cell adhesion glycoprotein encoded by the *CDH1* gene in humans, which has been characterized as a tumor suppressor [56,57]. Its loss of function is correlated with several solid tumors including ovarian and thought to contribute to tumor progression and metastasis [58].

It is important to emphasize that the lack of association between the selected SNPs in this study with ovarian cancer risk does not definitively answer this important question because additional change-of-function SNPs for these enzymes exist and should be explored further. Recent genetic studies have linked MPO to lung and ovarian cancers by demonstrating a striking correlation between the relative risk for development of the disease and the incidence of functionally distinct *MPO* polymorphisms [59]. Additionally, a SNP in NAD(P)H oxidase (rs4673) has been associated with increased risk of ovarian cancer [60]. In breast cancer, the presence of the *CAT* SNP (rs1001179), was shown to confer increased risk [24]. We have selected numerous additional SNPs based on their effect on enzyme activities or association with cancer. Several SNPs in *NOS2* have been associated with gastric, esophageal, skin and urogenital cancers [20,22]. Also, SNPs in *MnSOD*, *GPX1*, *GPX4*, *CAT* were found to be associated with prostate cancer [24].

Other studies have found a SNP in *MnSOD* (rs4880) and a SNP in *MPO* (rs2333227) to be associated with increased risk for ovarian cancer [34]. The *MPO* SNP we have analyzed in this study is in 100% concordance with SNP rs2333227 [61]. Thus, in addition to examining other changes of functional SNPs, increasing the size of our cohort may be sufficient to reach statistical significance in several of the SNPs chosen for this study. The strength of our study includes the comprehensive nature of redox genes studied and the translational aspect of our approach by assessing simultaneously clinical and genotypic characteristics of the population. We believe the fact that our control cohort is heterogeneous represents strength, because it includes patients considered at high risk for *BRCA1/2* mutation and those without any established risk factors for ovarian cancer, reflecting the baseline risk group (general population). Interestingly, patients who tested negative for *BRCA1/2* mutations, as well as, those with family history of *BRCA1/2* mutations but also tested negative for *BRCA1/2* should be considered at a higher risk profile than the general population. More importantly, to our knowledge, we are the first to report an association between the presence of this specific *CAT* SNP and ovarian cancer survival. The study has several limitations such as small sample size, the retrospective nature inherent to case-control studies, and the geographic restriction of the population. In our patient population, the recurrence rate was found to be 60.5%; however, the exact date of recurrence was not established making the computation of progression-free survival (PFS) impossible. We acknowledge that the determination of PFS would have strengthened our findings as PFS has often been used as a primary endpoint or a surrogate to overall survival in clinical trials [62–67].

It is now evident that oxidative stress plays a major role in the pathogenesis of cancer including ovarian cancer, however the exact mechanisms remain to be clarified. In this preliminary study we were able to show that a specific *CAT* SNP is associated with poor survival in ovarian cancer patients. Further studies examining other SNPs in key oxidants and antioxidant enzymes with higher number of patients will be needed to establish this link. SNPs in these enzymes may serve as potential markers for ovarian cancer, which are urgently needed. Our study indicates a strong association with the *CAT* SNP and survival of ovarian cancer patients, and thus may serve as a prognosticator.

## Supporting Information

S1 File Patient data and information. (PDF)
