# Supplementary material for: A Single Nucleotide Polymorphism in Catalase Is Strongly Associated with Ovarian Cancer Survival
Source: PLoS One. 2015 Aug 24;10(8):e0135739. doi: 10.1371/journal.pone.0135739 (PMC4547699; doi:10.1371/journal.pone.0135739)
Supplement: S1 File — (PDF) [file pone.0135739.s001.pdf]

Patient number	Family History of Breast Cancer	Family history of ovarian cancer	Stage	histology	Cancer recurrance	Family History of BRCA	Grade	Other Cancer	Lung Cancer	Colon Cancer	Blood Cancer	Prostate Cancer	Gastric Cancer	Skin Cancer	Pancreas Cancer
1	No	No	N/A	N/A	N/A	N/A	N/A	N/A	N/A	N/A	N/A	N/A	N/A	N/A	N/A
2	No	No	N/A	N/A	N/A	N/A	N/A	N/A	N/A	N/A	N/A	N/A	N/A	N/A	N/A
3	Yes	No	N/A	N/A	No	No	N/A	Lung	Yes	No	No	No	No	No	No
4	Yes	No	N/A	N/A	No	No	N/A	Lung	Yes	No	No	No	No	No	No
5	Yes	Yes	IIIC	Endometroid	Yes	No	High	No	No	No	No	No	No	No	No
6	Yes	No	N/A	N/A	N/A	No	N/A	LMS,Lung,Pancreas,Testes	Yes	No	No	No	No	No	Yes
7	Missing	Missing	Missing	Missing	Missing	Missing	Missing	Missing	Missing	Missing	Missing	Missing	Missing	Missing	Missing
8	Missing	Missing	Missing	Missing	Missing	Missing	Missing	Missing	Missing	Missing	Missing	Missing	Missing	Missing	Missing
9	No	No	N/A	N/A	N/A	N/A	N/A	N/A	N/A	N/A	N/A	N/A	N/A	N/A	N/A
10	Yes	Yes	IIIC	Serous	Yes	No	High	Gastric,	No	No	No	No	Yes	No	No
11	Yes	Yes	Missing	N/A	N/A	Yes	N/A	No	No	No	No	No	No	No	No
12	Yes	Yes	N/A	N/A	N/A	No	N/A	No	No	No	No	No	No	No	No
13	Yes	No	N/A	N/A	N/A	Yes	N/A	No	No	No	No	No	No	No	No
14	Missing	Missing	Missing	Missing	Missing	Missing	Missing	Missing	Missing	Missing	Missing	Missing	Missing	Missing	Missing
15	No	Yes	IIIC	Serous	No	No	High	Lung	Yes	No	No	No	No	No	No
16	Yes	No	N/A	N/A	N/A	No	N/A	Colon,Prostate	No	No	No	No	No	No	No
17	Missing	Missing	Missing	Missing	Missing	Missing	Missing	Missing	Missing	Missing	Missing	Missing	Missing	Missing	Missing
18	Yes	No	N/A	N/A	No	Yes	N/A	No	No	No	No	No	No	No	No
19	Yes	Yes	N/A	N/A		No	No	No	No	No	No	No	No	No	No
20	Yes	No	N/A	N/A	N/A	No	N/A	Gastric,Lung	Yes	No	No	No	Yes	No	No
21	Yes	No	N/A	N/A	N/A	No	N/A	No	No	No	No	No	No	No	No
22	Yes	No	N/A	N/A	N/A	No	N/A	Pancreas	No	No	No	No	No	No	Yes
23	No	No	N/A	N/A	N/A	N/A	N/A	N/A	N/A	N/A	N/A	N/A	N/A	N/A	N/A
24	Yes	Yes	N/A	N/A		Yes	N/A	Prostate,Kidney,Thyroid	No	No	No	Yes	No	No	No
25	Yes	Yes	N/A	N/A	N/A	No	N/A	No	No	No	No	No	No	No	No
26	Yes	No	N/A	N/A	Yes	No	High	No	No	No	No	No	No	No	No
27	Yes	Yes	IIIC	Serous	Yes	No	High	No	No	No	No	No	No	No	No
28	Yes	Yes	N/A	N/A	N/A	No	N/A	N/A	N/A	N/A	N/A	N/A	N/A	N/A	N/A
29	Missing	Missing	Missing	Missing	Missing	Missing	Missing	Missing	Missing	Missing	Missing	Missing	Missing	Missing	Missing
30	No	Yes	N/A	N/A	N/A	No	N/A	Colon	No	Yes	No	No	No	No	No
31	No	Yes			N/A	No	N/A	Colon	No	Yes	No	No	No	No	No
32	Yes	No	N/A	N/A	N/A	No	N/A	Colon, NHL	No	Yes	Yes	No	No	No	No
33	Yes	Yes	N/A	N/A	N/A	No	N/A	N/A	N/A	N/A	N/A	N/A	N/A	N/A	N/A
34	No	Yes	N/A	N/A	Yes	No	N/A	No	No	No	No	No	No	No	No
35	No	No	N/A	N/A	N/A	N/A	N/A	N/A	N/A	N/A	N/A	N/A	N/A	N/A	N/A
36	Yes	Yes	N/A	N/A	No	Yes	N/A	Thyroid	No	No	No	No	No	No	No
37	Yes	No	N/A	N/A	N/A	No	N/A	No	No	No	No	No	No	No	No
38	Yes	Yes	IIIC	serous	Yes	No	High	No	No	No	No	No	No	No	No

Patient number	Family History of Breast Cancer	Family history of ovarian cancer	Stage	histology	Cancer recurrance	Family History of BRCA	Grade	Other Cancer	Lung Cancer	Colon Cancer	Blood Cancer	Prostate Cancer	Gastric Cancer	Skin Cancer	Pancreas Cancer
39	Yes	Yes	N/A	N/A	N/A	No	N/A	Colon	No	Yes	No	No	No	No	No
40	Yes	Yes	?		N/A	No	N/A	Lung,Pancreas	Yes	No	No	No	No	No	Yes
41	MISSING	MISSING	MISSING	MISSING	MISSING	MISSING	MISSING	MISSING	MISSING	MISSING	MISSING	MISSING	MISSING	MISSING	MISSING
42	Yes	Yes	N/A	N/A	N/A	No	No	No	No	No	No	No	No	No	No
43	Yes	No	N/A	N/A	N/A	No	No	No	No	No	No	No	No	No	No
44	Yes	Yes	?	?	N/A	No	No	No	No	No	No	No	No	No	No
45	Yes	Yes	N/A	N/A	N/A	No	No	No	No	No	No	No	No	No	No
46	Yes	No	N/A	N/A	N/A	No	No	No	No	No	No	No	No	No	No
47	Yes	No	N/A	N/A	N/A	No	No	No	No	No	No	No	No	No	No
48	Yes	No	N/A	N/A	N/A	Yes	N/A	No	No	No	No	No	No	No	No
49	Yes	Yes	IV	Serous	?	No	No	No	No	No	No	No	No	No	No
50	No	Yes	IC	Serous	Yes	No	No	No	No	No	No	No	No	No	No
51	Yes	Yes	IV	Serous	No	Yes	High	Liver	No	No	No	No	No	No	No
52	Missing	Missing	Missing	Missing	Missing	Missing	Missing	Missing	Missing	Missing	Missing	Missing	Missing	Missing	Missing
53	No	Yes	IIIC	Serous	Yes	No	No	No	No	No	No	No	No	No	No
54	No	Yes	IIIC	Serous	Yes	No	No	No	No	No	No	No	No	No	No
55	Yes	Yes	N/A	N/A	N/A	Yes	N/A	Colon	No	Yes	No	No	No	No	No
56	No	No	N/A	N/A	N/A	N/A	N/A	N/A	N/A	N/A	N/A	N/A	N/A	N/A	N/A
57	No	Yes	?	N/A	N/A	Yes	N/A	No	No	No	No	No	No	No	No
58	Yes	Yes	N/A	N/A	N/A	No	N/A	No	No	No	No	No	No	No	No
59	No	Yes	IC	N/A	N/A	No	?	No	No	No	No	No	No	No	No
60	Yes	Yes	IIIC	Serous	Yes	No	High	No	No	No	No	No	No	No	No
61	Yes	No	N/A	N/A	No	No	N/A	No	No	No	No	No	No	No	No
62	Yes	Yes	IC	N/A	No	No	High	No	No	No	No	No	No	No	No
63	Yes	yes	IIIC	Serous	?	No	High	Gastric	No	No	No	No	Yes	No	No
64	Yes	yes	IIIC	Serous		No	High	Lung,prostate, colon	Yes	Yes	No	Yes	No	No	No
65	Yes	yes	IIIC	Serous	Yes	No	High	No	No	No	No	No	No	No	No
66	Yes	No	N/A	N/A	N/A	Yes	N/A	N/A	N/A	N/A	N/A	N/A	N/A	N/A	N/A
67	Yes	Yes	IIIB	Serous	No	No	?	Ovarian,sarcoma,gastric,prostate	No	No	No	Yes	Yes	No	No
68	No	No	N/A	N/A	N/A	N/A	N/A	N/A	N/A	N/A	N/A	N/A	N/A	N/A	N/A
69	Yes	No	N/A	N/A	N/A	No	N/A	Colon,esophagus		Yes	No	No	No	No	No
70	Yes	No	N/A	N/A	N/A	Yes	N/A	N/A	N/A	N/A	N/A	N/A	N/A	N/A	N/A
71	Yes	Yes	N/A	N/A	N/A	Yes	N/A	Prostate	No	No	No	Yes	No	No	No
72	Yes	No	N/A	N/A	N/A	Yes	N/A	No	No	No	No	No	No	No	No
73	Yes	No	N/A	N/A	N/A	Yes	N/A	No	No	No	No	No	No	No	No

Patient number	Family History of Breast Cancer	Family history of ovarian cancer	Stage	histology	Cancer recurrance	Family History of BRCA	Grade	Other Cancer	Lung Cancer	Colon Cancer	Blood Cancer	Prostate Cancer	Gastric Cancer	Skin Cancer	Pancreas Cancer
74	Yes	No	N/A	N/A	N/A	Yes	N/A	HL, Leukemia, Prostate,		No	Yes	Yes	No	No	No
75	Yes	Yes	N/A	N/A	N/A	Yes	N/A	Lung	Yes	No	No	No	No	No	No
76	Yes	Yes	IIA	serous	Yes	Yes	High	No	No	No	No	No	No	No	No
77	Yes	Yes	IIB	serous	Yes	No	High	Endometrial, colon		Yes	No	No	No	No	No
78	Yes	No	N/A	N/A	N/A	No	N/A	HEENT	No	No	No	No	No	No	No
79	Yes	Yes	IIIC	serous	No	No	High	No	No	No	No	No	No	No	No
80	Yes	Yes	IIA	Clear cell		No	?	Kidney, cervix, leukemia		No	Yes	No	No	No	No
81	Yes	Yes	IV	serous		No	High	Cervix, colon,leukemia ,brain		Yes	Yes	No	No	No	No
82	Yes	No	N/A	N/A	N/A	No	N/A	N/A	N/A	N/A	N/A	N/A	N/A	N/A	N/A
83	Yes	Yes	IIIC	serous	Yes	No	High	Lung,melanom a,colon	Yes	Yes	No	No	No	Yes	No
84	Yes	Yes	IIIC	serous	Yes	No	High	Lung, Leukemia	Yes	No	Yes	No	No	No	No
85	Yes	No	N/A	N/A	N/A	No	N/A	No	No	No	No	No	No	No	No
86	Yes	Yes	IIC	serous	No	No	N/A	Pancreas	No	No	No	No	No	No	Yes
87	Yes	Yes	I	serous	No	No	?	Pancreas, Prostate, Uterine, Skin		No	No	Yes	No	Yes	Yes
88	Yes	Yes	IC	Serous	No	No	High	Colon,ALL,MM	No	Yes	Yes	No	No	No	No
89	Yes	No	N/A	N/A	N/A	No	N/A	No	No	No	No	No	No	No	No
90	Yes	Yes	IIIC	serous	Yes	Yes	High	N/A	N/A	N/A	N/A	N/A	N/A	N/A	N/A
91	Yes	Yes	N/A	N/A	N/A	Yes	N/A	No	No	No	No	No	No	No	No
92	Yes	Yes	N/A	N/A	N/A	No	N/A	Bladder, Pancreas		No	No	No	No	No	Yes
93	Yes	Yes	IIIC	Serous	No	Yes	High	No	No	No	No	No	No	No	No
94	Yes	Yes	N/A	N/A	N/A	No	N/A	Gastric,pancre as	No	No	No	No	Yes	No	Yes
95	Yes	Yes	IIIC	serous	Yes	No	High	Gastric, colon	No	Yes	No	No	Yes	No	No
96	Yes	Yes	N/A	N/A	N/A	No	N/A	Lung	Yes	No	No	No	No	No	No
97	Yes	No	N/A	N/A	N/A	No	N/A	CLL	No	No	Yes	No	No	No	No
98	No	Yes	N/A	N/A	N/A	Yes	N/A	No	No	No	No	No	No	No	No
99	No	No	N/A	N/A	N/A	N/A	N/A	N/A	N/A	N/A	N/A	N/A	N/A	N/A	N/A
100	No	Yes	IIIC	serous	Yes	No	High	Lung, colon	Yes	Yes	No	No	No	No	No
101	Yes	Yes	N/A	N/A		No	N/A	Liver, Cervix, Brain, Spine		No	No	No	No	No	No

Patient number	Family History of Breast Cancer	Family history of ovarian cancer	Stage	histology	Cancer recurrance	Family History of BRCA	Grade	Other Cancer	Lung Cancer	Colon Cancer	Blood Cancer	Prostate Cancer	Gastric Cancer	Skin Cancer	Pancreas Cancer
102	Yes	Yes	Missing	Missing		No	missing	Cervix, Pancreas		No	No	No	No	No	Yes
103	Yes	Yes	N/A	N/A	N/A	No	N/A	Pancreas, Prostate		No	No	Yes	No	No	Yes
104	Yes	Yes	N/A	N/A	N/A	No	N/A	Colon, Prostate,MM		Yes	Yes	Yes	No	No	No
105	No	Yes	IIIC	serous	Yes	No	High	Colon,Bladder, Lung,Uterus, Skin	Yes	Yes	No	No	No	Yes	No
106	Yes	Yes	N/A	N/A	N/A	No	N/A	Colon, Lung, HEENT, Bladder	Yes	Yes	Yes	No	No	No	No
107	Yes	No	N/A	N/A	N/A	No	N/A	Prostate, thyroid,cervix, HEENT	No	No	No	Yes	No	No	
108	Yes	No	N/A	N/A	N/A	No	N/A	Prostate, thyroid,cervix, HEENT,skin	No	No	No	Yes	No	Yes	
109	Yes	Yes	N/A	N/A	N/A	No	N/A	Prostate, skin, thyroid,HEENT, Gastric, Blood,, cervix			Yes	Yes	Yes	Yes	No
110	Yes	No	N/A	N/A	N/A	No	N/A	Prostate, BCC_skin, thyroid,HEENT, Gastric, Blood,			Yes	No	Yes	Yes	No
111	Yes	No	N/A	N/A	N/A	No	N/A	Lymph	No	No	Yes	No	No	No	No
112	Yes	Yes	N/A	N/A	N/A	No	N/A	Endometrial,Lung, Kidney,HL,	Yes	No	No	No	No	No	No
113	Yes	Yes	N/A	N/A	N/A	No	N/A	Colon,Lymph, cervix		Yes	Yes	No	No	No	No
114	Yes	Yes	N/A	N/A	N/A	Yes	N/A	Colon,Lung	Yes	Yes	No	No	No	No	No
115	Yes	No	N/A	N/A	N/A	No	N/A	No	No	No	No	No	No	No	No
116															
117	No	Yes	IIIC	Serous	Yes	No	High	No	No	No	No	No	No	No	No
118	Yes	Yes	N/A	N/A	N/A	No	N/A	No	No	No	No	No	No	No	No
119	Yes	Yes	N/A	N/A	N/A	No	N/A	No	No	No	No	No	No	No	No
120	Yes	No	N/A	N/A	Lung, gastric,	No	N/A	No	No	No	No	No	No	No	No
121	Yes	No	N/A	N/A	N/A	Yes	N/A	No	No	No	No	No	No	No	No
122	Yes	No	N/A	N/A	N/A	No	N/A	Lung, Skin, Liver	Yes	No	No	No	No	Yes	No

Patient number	Family History of Breast	Family history of ovarian	Stage	histology	Cancer recurrance	Family History of BRCA	Grade	Other Cancer	Lung Cancer	Colon Cancer	Blood Cancer	Prostate Cancer	Gastric Cancer	Skin Cancer	Pancreas Cancer
123	Yes	No	N/A	N/A	N/A	No	N/A	Lung, Skin, Liver	Yes	No	No	No	No	Yes	No
124	Yes	Yes	N/A	N/A	N/A	No	N/A	Colon,Prostate ,Gastric, Skin		Yes	No	Yes	Yes	Yes	No
125	Yes	Yes	IIIC	serous	Yes	No	High	Prostate, Blood,colon, cervix		Yes	Yes	Yes	No	No	No
126	Yes	Yes	N/A	N/A	N/A	No	N/A	No	No	No	No	No	No	No	No
127	Yes	Yes	IIIC	serous	Yes	No	High	Prostate, lung		No	No	No	No	No	No
128	Yes	Yes	N/A	N/A	N/A	No	N/A	BCC_skin, Bladder,Brain, Gastric, Blood			Yes	No	Yes	Yes	No
129	Yes	Yes	N/A	serous		No	N/A	Colon,Prostate ,Lung,Gastric	Yes	Yes	No	Yes	Yes	No	No
130	Yes	Yes	N/A	serous		No	N/A	Skin, SCC, Endometrial,bo rderline			No	No	No	Yes	No
131	Yes	Yes	IIIC	serous	Yes	No	High	Prostate, lung,gastric, skin,	Yes	No	No	Yes	Yes	Yes	No
132	Yes	Yes	N/A	N/A	N/A	No	N/A	No	No	No	No	No	No	No	No
133	Yes	Yes	IIIC	serous	Yes	No	High	NHL, pancreas,BCC	No	No	Yes	No	No	Yes	Yes
134	Yes	Yes	IIIC	serous	Yes	No	High	Colon	No	Yes	No	No	No	No	No
135	No	No	N/A	N/A	N/A	N/A	N/A	N/A	N/A	N/A	N/A	N/A	N/A	N/A	N/A
136	No	No	N/A	N/A	N/A	N/A	N/A	N/A	N/A	N/A	N/A	N/A	N/A	N/A	N/A
137	No	No	N/A	N/A	N/A	N/A	N/A	N/A	N/A	N/A	N/A	N/A	N/A	N/A	N/A
138	No	No	N/A	N/A	N/A	N/A	N/A	N/A	N/A	N/A	N/A	N/A	N/A	N/A	N/A
139	No	No	N/A	N/A	N/A	N/A	N/A	N/A	N/A	N/A	N/A	N/A	N/A	N/A	N/A
140	No	No	N/A	N/A	N/A	N/A	N/A	N/A	N/A	N/A	N/A	N/A	N/A	N/A	N/A
141	No	No	N/A	N/A	N/A	N/A	N/A	N/A	N/A	N/A	N/A	N/A	N/A	N/A	N/A
142	No	No	N/A	N/A	N/A	N/A	N/A	N/A	N/A	N/A	N/A	N/A	N/A	N/A	N/A
143	No	No	N/A	N/A	N/A	N/A	N/A	N/A	N/A	N/A	N/A	N/A	N/A	N/A	N/A
144	No	No	N/A	N/A	N/A	N/A	N/A	N/A	N/A	N/A	N/A	N/A	N/A	N/A	N/A

Cervix Cancer	Uterus Cancer	Thyroid Cancer	Bladder Cancer	Kidney Cancer	Liver Cancer	Testes Cancer	Sarcoma Cancer	Esophagous Cancer	Spine Cancer	Brain Cancer	Head&Neck Cancer	rs1001179 (CAT)	rs1002149 (GSR)	rs11549465 (HIF1a)	rs2243828 (MPO)
N/A	N/A	N/A	N/A	N/A	N/A	N/A	N/A	N/A	N/A	N/A	N/A	C/C	G/G	C/C	A/A
N/A	N/A	N/A	N/A	N/A	N/A	N/A	N/A	N/A	N/A	N/A	N/A	C/T	G/G	C/C	A/G
No	No	No	No	No	No	No	No	No	No	No	No	C/C	G/T	C/T	A/A
No	No	No	No	No	No	No	No	No	No	No	No	C/C	UND	T/T	A/A
No	No	No	No	No	No	No	No	No	No	No	No	C/C	G/G	C/C	A/A
No	No	No	No	No	No	No	No	No	No	No	No	C/T	G/T	C/C	A/A
Missing	Missing	Missing	Missing	Missing	Missing	Missing	Missing	Missing	Missing	Missing	Missing	C/C	G/G	C/C	A/G
Missing	Missing	Missing	Missing	Missing	Missing	Missing	Missing	Missing	Missing	Missing	Missing	C/C	UND	C/C	A/A
N/A	N/A	N/A	N/A	N/A	N/A	N/A	N/A	N/A	N/A	N/A	N/A	C/C	G/G	C/C	A/G
No	No	No	No	No	No	No	No	No	No	No	No	C/T	UND	C/C	A/A
No	No	No	No	No	No	No	No	No	No	No	No	C/C	G/T	C/T	A/A
No	No	No	No	No	No	No	No	No	No	No	No	C/T	G/G	C/C	A/A
No	No	No	No	No	No	No	No	No	No	No	No	C/C	G/G	C/C	A/A
Missing	Missing	Missing	Missing	Missing	Missing	Missing	Missing	Missing	Missing	No	No	C/T	G/G	C/C	A/A
No	No	No	No	No	No	No	No	No	No	No	No	C/T	G/G	C/C	A/G
No	No	No	No	No	No	No	No	No	No	No	No	C/C	G/G	C/C	A/G
Missing	Missing	Missing	Missing	No	No	No	No	No	No	No	No	C/C	G/G	C/C	A/A
No	No	No	No	No	No	No	No	No	No	No	No	C/T	G/G	C/C	A/A
No	No	No	No	No	No	No	No	No	No	No	No	C/T	G/G	C/T	A/A
No	No	No	No	No	No	No	No	No	No	No	No	C/T	G/T	C/C	A/G
No	No	No	No	No	No	No	No	No	No	No	No	C/C	G/T	C/C	A/A
No	No	No	No	No	No	No	No	No	No	No	No	C/C	G/G	C/C	A/A
N/A	N/A	N/A	N/A	N/A	N/A	N/A	N/A	N/A	N/A	N/A	N/A	C/C	G/G	C/T	A/G
No	No	Yes	No	yes	No	No	No	No	No	No	No	C/C	G/T	C/C	G/G
No	No	No	No	No	No	No	No	No	No	No	No	C/C	G/G	C/C	A/A
No	No	No	No	No	No	No	No	No	No	No	No	C/C	G/G	C/C	A/A
No	No	No	No	No	No	No	No	No	No	No	No	C/T	G/G	C/C	A/A
N/A	N/A	N/A	N/A	N/A	N/A	N/A	N/A	N/A	N/A	No	No	C/T	G/G	C/T	A/A
Missing	Missing	Missing	Missing	Missing	Missing	Missing	Missing	Missing	Missing	No	No	C/C	G/G	C/C	A/A
No	No	No	No	No	No	No	No	No	No	No	No	C/C	G/T	C/C	A/A
No	No	No	No	No	No	No	No	No	No	No	No	C/C	G/G	C/C	A/A
No	No	No	No	No	No	No	No	No	No	No	No	C/T	G/G	C/T	A/G
N/A	N/A	N/A	N/A	N/A	N/A	N/A	N/A	N/A	N/A	No	No	C/C	G/T	C/C	A/A
No	No	No	No	No	No	No	No	No	No	No	No	C/C	UND	C/C	UND
N/A	N/A	N/A	N/A	N/A	N/A	N/A	N/A	N/A	N/A	N/A	N/A	C/C	G/G	C/C	A/A
No		Yes	Yes	Yes	Yes	Yes	Yes	Yes	Yes	No	No	C/C	G/T	C/C	A/G
No	No	No	No	No	No	No	No	No	No	No	No	C/C	G/G	C/C	A/A
No	No	No	No	No	No	No	No	No	No	No	No	C/T	G/G	C/C	G/G

Cervix Cancer	Uterus Cancer	Thyroid Cancer	Bladder Cancer	Kidney Cancer	Liver Cancer	Testes Cancer	Sarcoma Cancer	Esophagous Cancer	Spine Cancer	Brain Cancer	Head&Neck Cancer	rs1001179 (CAT)	rs1002149 (GSR)	rs11549465 (HIF1a)	rs2243828 (MPO)
No	No	No	No	No	No	No	No	No	No	No	No	C/T	G/G	C/C	A/G
No	No	No	No	No	No	No	No	No	No	No	No	C/T	G/T	C/C	A/G
MISSING	MISSING	MISSING	MISSING	MISSING	MISSING	MISSING	MISSING	MISSING	MISSING	No	No	C/C	G/G	C/C	A/A
No	No	No	No	No	No	No	No	No	No	No	No	C/C	G/G	C/C	A/A
No	No	No	No	No	No	No	No	No	No	No	No	C/C	G/G	C/C	A/A
No	No	No	No	No	No	No	No	No	No	Yes	No	C/C	G/G	C/C	A/G
No	No	No	No	No	No	No	No	No	No	Yes	No	C/C	G/T	C/C	A/A
No	No	No	No	No	No	No	No	No	No	No	No	C/C	G/G	C/C	A/A
No	No	No	No	No	No	No	No	No	No	No	No	C/T	G/G	C/C	A/A
No	No	No	No	No	No	No	No	No	No	No	No	C/T	G/G	C/C	A/A
No	No	No	No	No	No	No	No	No	No	No	No	C/C	UND	C/C	UND
No	No	No	No	No	No	No	No	No	No	No	No	C/C	UND	C/T	UND
No	No	No	No	No	Yes	No	No	No	No	No	No	C/C	G/G	C/C	A/G
Missing	Missing	Missing	Missing	Missing	Missing	Missing	Missing	Missing	Missing	Missing	Missing	C/C	G/G	C/T	A/A
No	No	No	No	No	No	No	No	No	No	No	No	C/C	G/T	C/C	A/G
No	No	No	No	No	No	No	No	No	No	No	No	C/C	G/T	C/C	A/G
No	No	No	No	No	No	No	No	No	No	No	No	C/C	G/G	C/C	A/A
N/A	N/A	N/A	N/A	N/A	N/A	N/A	N/A	N/A	N/A	N/A	N/A	C/C	G/G	C/C	A/A
No	No	No	No	No	No	No	No	No	No	No	No	C/T	G/G	C/T	A/G
No	No	No	No	No	No	No	No	No	No	No	No	C/C	G/G	C/C	A/A
No	No	No	No	No	No	No	No	No	No	No	No	C/T	G/G	C/T	A/G
No	No	No	No	No	No	No	No	No	No	No	No	T/T	G/G	C/C	A/G
No	No	No	No	No	No	No	No	No	No	No	No	C/C	T/T	C/C	A/A
No	No	No	No	No	No	No	No	No	No	No	No	C/C	G/G	C/C	A/A
No	No	No	No	No	No	No	No	No	No	No	No	C/C	G/G	C/C	A/A
No	No	No	No	No	No	No	No	No	No	No	No	C/C	G/T	C/C	A/A
No	No	No	No	No	No	No	No	No	No	No	No	C/T	G/G	C/T	A/A
N/A	No	N/A	N/A	N/A	N/A	N/A	N/A	N/A	N/A	N/A	N/A	C/T	G/G	C/C	G/G
No	No	No	No	No	No	No	Yes	No	No	No	No	C/C	T/T	C/C	A/A
N/A	N/A	N/A	N/A	N/A	N/A	N/A	N/A	N/A	N/A	N/A	N/A	C/C	G/G	C/C	A/A
No	No	No	No	No	No	No	No	No	No	No	No	C/C	G/G	C/T	A/A
N/A		No	No	No	No	No	No	No	No	No	No	C/T	G/T	C/T	A/A
No	No	No	No	No	No	No	No	No	No	No	No	C/C	G/G	C/C	A/A
No	No	No	No	No	No	No	No	No	No	No	No	C/T	G/T	C/C	A/A
No	No	No	No	No	No	No	No	No	No	No	No	C/C	G/G	C/C	G/G

Cervix Cancer	Uterus Cancer	Thyroid Cancer	Bladder Cancer	Kidney Cancer	Liver Cancer	Testes Cancer	Sarcoma Cancer	Esophagous Cancer	Spine Cancer	Brain Cancer	Head&Neck Cancer	rs1001179 (CAT)	rs1002149 (GSR)	rs11549465 (HIF1a)	rs2243828 (MPO)
No	No	No	No	No	No	No	No	No	No	No	No	C/T	T/T	C/T	G/G
No	No	No	No	No	No	No	No	No	No	No	No	C/T	G/G	C/C	A/G
No	No	No	No	No	No	No	No	No	No	No	No	C/T	G/T	C/C	A/A
No	Yes	No	No	No	No	No	No	No	No	No	No	C/T	G/G	C/C	G/G
No	No	No	No	No	No	No	No	No	No	No	Yes	C/C	G/G	C/C	A/A
No	No	No	No	No	No	No	No	No	No	No	No	C/T	G/G	C/C	A/A
Yes	No	No	No	Yes	No	No	No	No	No	No	No	C/C	G/T	C/C	A/G
Yes	No	No	No	No	No	No	No	No	No	Yes	No	C/C	G/G	C/C	A/G
N/A	N/A	N/A	No	No	No	No	No	No	No	No	No	C/T	G/G	C/C	G/G
No	No	No	No	No	No	No	No	No	No	No	No	C/T	G/T	C/T	A/G
No	No	No	No	No	No	No	No	No	No	No	No	C/T	G/T	C/T	G/G
No	No	No	No	No	No	No	No	No	No	No	No	C/T	G/G	C/C	A/G
No	No	No	No	No	No	No	No	No	No	No	No	C/C	G/G	C/T	A/A
No	Yes	No	No	No	No	No	No	No	No	No	No	C/C	G/G	C/C	G/G
No	No	No	No	No	No	No	No	No	No	No	No	C/C	G/G	C/C	A/A
No	No	No	No	No	No	No	No	No	No	No	No	C/C	G/G	C/C	A/A
N/A	N/A	N/A	N/A	No	No	No	No	No	No	No	No	C/T	G/G	C/C	A/G
No	No	No	No	No	No	No	No	No	No	No	No	C/C	G/G	C/C	A/G
			Yes	No	No	No	No	No	No	No	No	C/C	G/T	C/C	A/G
No	No	No	No	No	No	No	No	No	No	No	No	C/T	G/G	C/C	A/A
		No	No	No	No	No	No	No	No	No	No	C/C	G/G	C/T	A/G
No	No	No	No	No	No	No	No	No	No	No	No	T/T	G/T	C/C	A/G
No	No	No	No	No	No	No	No	No	No	No	No	C/C	G/G	C/C	A/A
No	No	No	No	No	No	No	No	No	No	No	No	C/C	G/G	C/C	A/A
No	No	No	No	No	No	No	No	No	No	No	No	C/C	G/G	C/C	A/A
N/A	N/A	N/A	N/A	N/A	N/A	N/A	N/A	N/A	N/A	N/A	N/A	C/T	G/G	C/C	A/A
No	No	No	No	No	No	No	No	No	No	No	No	C/C	G/G	C/C	A/A
Yes	No	No	No	No	Yes	No	No	No	Yes	Yes	No	C/C	G/G	C/C	A/G

Cervix Cancer	Uterus Cancer	Thyroid Cancer	Bladder Cancer	Kidney Cancer	Liver Cancer	Testes Cancer	Sarcoma Cancer	Esophagous Cancer	Spine Cancer	Brain Cancer	Head&Neck Cancer	rs1001179 (CAT)	rs1002149 (GSR)	rs11549465 (HIF1a)	rs2243828 (MPO)
Yes	No	No	No	No	No	No	No	No	No	No	No	C/C	G/G	C/C	A/A
No	No	No	No	No	No	No	No	No	No	No	No	C/C	G/G	C/T	A/G
No	No	No	No	No	No	No	No	No	No	No	No	C/T	G/G	C/C	A/A
No	Yes	No	Yes	No	No	No	No	No	No	No	No	C/T	G/T	C/C	A/A
No	Yes	No	Yes	No	No	No	No	No	No	No	Yes	C/T	G/G	C/C	A/A
Yes		No	No	No	No	No	No	No	No	No	Yes	C/C	G/G	C/T	A/G
Yes		No	No	No	No	No	No	No	No	No	Yes	C/C	G/G	C/C	A/A
Yes	No	No	No	No	No	No	No	No	No	No	Yes	C/C	G/G	T/T	A/G
No	No	Yes	Yes	No	No	No	No	No	No	No	Yes	C/T	G/G	C/C	A/G
		No	No	No	No	No	No	No	No	No	No	C/C	UND	C/C	A/A
No	No	No	No	Yes	No	No	No	No	No	No	No	C/C	G/T	C/C	A/A
Yes	No	No	No	No	No	No	No	No	No	No	No	C/C	G/T	C/T	A/A
No	No	No	No	No	No	No	No	No	No	No	No	C/C	G/G	C/C	A/G
No	No	No	No	No	No	No	No	No	No	No	No	C/T	G/G	C/C	A/G
No	No	No	No	No	No	No	No	No	No	No	No	C/C	G/T	C/C	G/G
No	No	No	No	No	No	No	No	No	No	No	No	C/C	G/G	C/C	A/A
No	No	No	No	No	No	No	No	No	No	No	No	C/C	G/G	C/C	A/A
No	No	No	No	No	No	No	No	No	No	No	No	C/C	G/G	C/C	A/G
No	No	No	No	No	No	No	No	No	No	No	No	C/T	G/G	C/C	A/G
No	No	No	No	No	Yes	No	No	No	No	No	No	C/C	G/T	C/C	A/G

Cervix Cancer	Uterus Cancer	Thyroid Cancer	Bladder Cancer	Kidney Cancer	Liver Cancer	Testes Cancer	Sarcoma Cancer	Esophagous Cancer	Spine Cancer	Brain Cancer	Head&Neck Cancer	rs1001179 (CAT)	rs1002149 (GSR)	rs11549465 (HIF1a)	rs2243828 (MPO)
No	No	No	No	No	Yes	No	No	No	No	No	No	C/T	G/G	C/C	A/G
No	No	No	No	No	No	No	No	No	No	No	No	C/C	G/T	C/C	A/A
Yes	No	No	No	No	No	No	No	No	No	No	No	C/C	G/T	C/C	A/A
No	No	No	No	No	No	No	No	No	No	No	No	C/C	G/G	C/C	A/G
No	No	No	No	No	No	No	No	No	No	No	No	C/C	G/G	C/C	A/A
No	No	No	No	No	No	No	No	No	No	Yes	No	C/C	G/T	C/C	A/G
No	No	No	No	No	No	No	No	No	No	No	No	C/C	G/G	C/T	G/G
No	Yes	No	No	No	No	No	No	No	No	No	No	C/C	G/G	C/C	A/A
No	No	No	No	No	No	No	No	No	No	No	No	C/T	G/T	C/T	A/G
No	No	No	No	No	No	No	No	No	No	No	No	C/C	G/G	C/C	A/A
No	No	No	No	No	No	No	No	No	No	No	No	T/T	UND	C/C	G/G
No	No	No	No	No	No	No	No	No	No	No	No	C/T	G/G	C/C	A/A
N/A	N/A	N/A	N/A	N/A	N/A	N/A	N/A	N/A	N/A	N/A	N/A	C/C	G/G	C/C	A/A
N/A	N/A	N/A	N/A	N/A	N/A	N/A	N/A	N/A	N/A	N/A	N/A	C/C	G/G	C/T	A/A
N/A	N/A	N/A	N/A	N/A	N/A	N/A	N/A	N/A	N/A	N/A	N/A	C/T	G/G	C/C	A/A
N/A	N/A	N/A	N/A	N/A	N/A	N/A	N/A	N/A	N/A	N/A	N/A	C/T	G/G	C/C	A/A
N/A	N/A	N/A	N/A	N/A	N/A	N/A	N/A	N/A	N/A	N/A	N/A	C/C	G/T	C/C	A/G
N/A	N/A	N/A	N/A	N/A	N/A	N/A	N/A	N/A	N/A	N/A	N/A	C/C	G/G	C/T	A/A
N/A	N/A	N/A	N/A	N/A	N/A	N/A	N/A	N/A	N/A	N/A	N/A	C/C	G/G	C/C	A/A
N/A	N/A	N/A	N/A	N/A	N/A	N/A	N/A	N/A	N/A	N/A	N/A	C/C	G/T	C/C	A/A
N/A	N/A	N/A	N/A	N/A	N/A	N/A	N/A	N/A	N/A	N/A	N/A	C/C	G/G	C/C	A/A
N/A	N/A	N/A	N/A	N/A	N/A	N/A	N/A	N/A	N/A	N/A	N/A	C/C	G/G	C/C	A/G

rs2297518 (iNOS)	rs3448 (GPX1)	rs4673 (CYBA)	rs4880 (MnSOD)	Survival in months
G/G	C/C	A/G	T/C	missing!
A/G	C/T	G/G	T/T	missing!
G/G	T/T	T/C	A/G	missing!
A/G	T/T	T/T	G/G	missing!
G/G	C/C	T/T	G/G	288.00
G/G	C/C	C/C	G/G	missing!
G/G	C/T	UND	A/G	missing!
A/A	C/C	C/C	A/G	missing!
G/G	C/T	G/G	T/C	missing!
G/G	C/C	T/T	A/G	60.00
A/G	C/C	C/C	G/G	missing!
G/G	C/C	T/C	A/A	missing!
G/G	C/T	T/C	A/G	missing!
A/G	C/C	T/C	A/G	missing!
G/G	T/T	T/T	A/A	60.00
G/G	T/T	T/C	A/G	missing!
G/G	C/T	T/C	G/G	missing!
G/G	C/C	T/C	A/G	missing!
G/G	C/C	T/T	A/G	missing!
G/G	C/C	T/T	A/A	missing!
G/G	C/T	C/C	A/G	missing!
G/G	C/T	T/T	A/A	missing!
G/G	C/C	A/G	T/T	missing!
G/G	C/T	T/T	A/A	missing!
G/G	C/C	T/C	G/G	missing!
A/A	C/C	T/C	A/G	missing!
A/G	C/C	C/C	A/G	missing!
A/G	C/T	T/T	G/G	missing!
G/G	C/T	T/C	A/A	missing!
A/G	C/T	C/C	A/G	708.00
G/G	C/C	T/C	A/G	missing!
A/G	C/C	T/T	G/G	missing!
G/G	C/T	T/T	A/A	12.00
UND	UND	UND	A/A	96.00
A/G	C/C	G/G	T/C	missing!
G/G	C/T	T/C	G/G	missing!
G/G	C/T	T/T	A/A	missing!
A/G	C/T	C/C	G/G	12.00

rs2297518 (iNOS)	rs3448 (GPX1)	rs4673 (CYBA)	rs4880 (MnSOD)	Survival in months
G/G	C/C	T/T	G/G	missing!
G/G	C/T	T/C	A/G	396.00
G/G	C/C	T/T	A/G	missing!
G/G	C/T	T/C	A/G	missing!
G/G	C/C	T/C	A/G	missing!
A/G	C/C	T/C	G/G	missing!
G/G	C/C	C/C	A/G	missing!
G/G	C/C	C/C	A/G	missing!
A/A	C/C	T/T	A/G	missing!
G/G	C/T	T/C	A/A	missing!
UND	UND	UND	G/G	300.00
G/G	C/T	T/T	A/G	missing!
G/G	C/C	T/T	A/G	108.00
G/G	C/C	T/C	A/G	missing!
G/G	C/T	C/C	G/G	108.00
G/G	C/C	C/C	A/G	36.00
A/G	C/C	T/T	A/A	missing!
A/G	C/C	A/G	T/C	missing!
A/A	C/T	T/C	G/G	missing!
A/G	C/C	C/C	A/A	missing!
G/G	C/C	T/C	G/G	missing!
G/G	C/C	C/C	A/G	60.00
A/G	C/C	T/T	G/G	missing!
G/G	C/C	C/C	G/G	missing!
G/G	C/C	T/C	G/G	24.00
G/G	C/C	T/T	A/G	missing!
A/G	C/C	T/C	G/G	missing!
G/G	C/C	C/C	A/G	missing!
G/G	C/T	C/C	A/G	missing!
A/G	C/T	A/G	T/C	missing!
G/G	C/C	T/T	A/A	missing!
G/G	C/C	T/C	A/G	missing!
G/G	C/C	T/T	G/G	missing!
G/G	C/C	T/C	A/G	missing!
G/G	C/T	T/C	A/G	missing!

rs2297518 (iNOS)	rs3448 (GPX1)	rs4673 (CYBA)	rs4880 (MnSOD)	Survival in months
A/G	C/C	C/C	G/G	missing!
G/G	C/C	T/C	G/G	missing!
G/G	C/C	T/C	G/G	missing!
A/A	C/C	T/C	G/G	missing!
G/G	T/T	T/T	A/A	missing!
A/G	C/T	T/T	A/G	72.00
G/G	C/T	T/C	G/G	missing!
A/G	T/T	T/T	G/G	48.00
A/A	C/T	T/C	G/G	missing!
A/G	C/T	T/C	G/G	48.00
A/G	C/C	T/C	G/G	missing!
G/G	C/C	T/C	A/A	missing!
G/G	C/C	C/C	G/G	missing!
A/G	C/C	T/C	A/G	missing!
A/G	C/C	T/C	G/G	missing!
G/G	C/T	T/T	G/G	missing!
A/G	C/C	C/C	G/G	108.00
G/G	C/T	C/C	A/G	missing!
G/G	C/C	T/C	A/A	missing!
G/G	C/T	T/C	A/G	72.00
G/G	C/C	C/C	A/G	missing!
A/G	C/C	T/C	G/G	60.00
G/G	C/T	T/C	A/G	missing!
G/G	C/T	T/C	A/G	missing!
A/G	C/C	C/C	A/G	missing!
G/G	C/C	G/G	T/C	missing!
G/G	C/C	T/C	A/G	missing!
G/G	C/C	C/C	A/A	missing!

rs2297518 (iNOS)	rs3448 (GPX1)	rs4673 (CYBA)	rs4880 (MnSOD)	Survival in months
G/G	C/C	T/C	A/G	missing!
A/G	C/C	T/C	A/A	missing!
A/G	C/T	C/C	A/G	missing!
G/G	C/T	C/C	A/G	missing!
G/G	T/T	T/T	A/G	missing!
A/G	C/T	C/C	G/G	missing!
A/G	C/T	C/C	A/G	missing!
A/G	C/T	C/C	A/G	missing!
A/A	C/C	T/C	A/A	missing!
G/G	C/C	C/C	A/G	missing!
G/G	C/T	C/C	A/G	missing!
G/G	C/C	T/C	G/G	missing!
G/G	C/T	T/T	A/G	missing!
A/G	C/C	T/C	A/A	missing!
G/G	C/T	T/C	A/G	72.00
G/G	C/C	T/T	A/G	missing!
G/G	C/C	T/T	A/A	missing!
G/G	C/T	T/C	A/G	missing!
A/G	C/C	C/C	G/G	missing!
G/G	C/C	T/T	G/G	missing!

rs2297518 (iNOS)	rs3448 (GPX1)	rs4673 (CYBA)	rs4880 (MnSOD)	Survival in months
A/G	C/C	C/C	G/G	missing!
UND	C/C	T/T	A/A	missing!
A/G	C/T	T/C	A/G	missing!
A/G	C/T	T/T	A/G	missing!
G/G	C/C	T/C	A/G	missing!
G/G	C/C	T/C	G/G	missing!
G/G	C/T	C/C	G/G	missing!
G/G	T/T	T/T	G/G	missing!
G/G	C/T	T/C	A/G	missing!
A/G	C/T	T/T	G/G	missing!
A/G	C/T	T/C	A/G	missing!
G/G	C/T	C/C	A/G	36.00
G/G	C/C	A/A	C/C	missing!
G/G	C/C	A/G	T/T	missing!
G/G	C/T	A/G	T/C	missing!
G/G	C/C	A/G	T/C	missing!
G/G	C/T	A/G	T/C	missing!
G/G	C/T	A/G	T/C	missing!
A/G	T/T	G/G	C/C	missing!
G/G	C/C	G/G	T/T	missing!
A/G	C/C	A/G	C/C	missing!
A/G	C/C	G/G	T/T	missing!
